# Biofortification: Effect of Iodine Fortified Food in the Healthy Population, Double-Arm Nutritional Study

**DOI:** 10.3389/fnut.2022.871638

**Published:** 2022-03-23

**Authors:** Sara Baldassano, Francesca Di Gaudio, Leo Sabatino, Rosalia Caldarella, Claudio De Pasquale, Luigi Di Rosa, Domenico Nuzzo, Pasquale Picone, Sonya Vasto

**Affiliations:** ^1^Department of Biological, Chemical and Pharmaceutical Sciences and Technologies, University of Palermo, Palermo, Italy; ^2^Department of Promoting Health, Maternal-Infant, Excellence and Internal and Specialized Medicine (ProMISE) G. D’Alessandro, University of Palermo, Palermo, Italy; ^3^Dipartimento Scienze Agrarie, Alimentari e Forestali (SAAF), University of Palermo, Palermo, Italy; ^4^Department of Laboratory Medicine, “P. Giaccone” University Hospital, Palermo, Italy; ^5^Istituto per la Ricerca e l’Innovazione Biomedica (IRIB), CNR, Palermo, Italy; ^6^Euro-Mediterranean Institutes of Science and Technology (IEMEST), Palermo, Italy

**Keywords:** biofortification, iodine, vitamin D, calcium, functional food

## Abstract

It is estimated that one-third of the world’s population lives in areas where iodine (I) is scarce and its deficiency is responsible for many related disorders, such as goiter, reproductive failure, hearing loss, growth impairment, congenital I deficiency syndrome, and numerous kinds of brain injury. Mineral deficiencies can be overcome *via* dietary diversification and mineral supplementation. An alternative or even complementary way is represented by the intake of biofortified foods, which can tackle this lack of micronutrients. In this short-term double-arm nutritional intervention study, a cohort of ten people was supplemented with curly endive leaf biofortified with I and ten people with curly endive without biofortification (Intervention Study on Iodine Biofortification Vegetables (Nutri-I-Food – Full-Text View - ClinicalTrials.gov). The effects on whole-body homeostasis and specifically on I, glucose, lipid, and hepatic, iron metabolism was investigated. Blood samples were obtained at baseline and after 12 days of supplementation with curly endive and compared with controls. Hematochemical and urinary parameters were analyzed at baseline and after 12 days. The results showed that short-term I curly endive intervention did not affect the whole body homeostasis in healthy people and revealed an increase in I concentration in urine samples and an increase in vitamin D, calcium, and potassium concentration in blood samples only in the biofortified cohort respect to controls. This study suggests that short-term consumption of I curly endive crops is safe and could positively impact body health.

## Introduction

Globally, 2 billion people suffer from micronutrient deficiency that strongly impacts their health status. In relation to iodine (I), it is estimated that one-third of the world’s population lives in areas where I is scarce and its deficiency is responsible for many related disorders, such as goiter, reproductive failure, hearing loss, growth impairment, congenital I deficiency syndrome, and numerous kinds of brain injury (1–4). It is quite clear that mineral deficiencies can be overcome through careful dietary diversification and mineral supplementation. An alternative or even complementary way is represented by the intake of biofortified foods, which can tackle this lack of micronutrients. Biofortification is well documented as a possible strategy, from the economic and environmental point of view, to fight mineral malnourishment in several populations (5, 6).

Furthermore, since trace elements might be indispensable for humans, but beneficial for plants at mild dosages, a number of studies were conducted to find adequate methods of application and optimal dose in vegetable crops (7–14).

In 2007, the World Health Organization (WHO) proposed a different way to increase I intake *via* the consumption of seafood and biofortified food, such as fruiting and leafy green vegetables. In this respect, several studies aimed to I-enrichment in various fruiting and leafy vegetable crops, such as lettuce, spinach, and tomato (5, 14–18). In particular, for I ingestion, the European Food Safety Authority (EFSA NDA Panel, 2014) suggested a daily dosage of 90–120 μg for kids, 150 μg for adult men or women, and 290 μg for prenatal or lactating women (19).

In literature, the iodination of table salt comes as the first approach to overcome I deficiency (20) although critics pointed out that salt iodination alone is unsatisfactory to cover up the full requirement of I in human health (21). This is probably due to the volatile aspect of inorganic I and its loss during life shelf, transport, and cooking. Furthermore, the use of table salt is not advised for people affected by cardiovascular disease although its influence is still a matter of debate (22); nonetheless, some literature shows a clear association between salt intake and blood pressure (23).

Curly endive (*Cichorium endivia L. var. crispum Hegi*) is extensively cultivated worldwide and appreciated for direct consumption or as an ingredient of mixed ready-to-eat salads. To the best of our knowledge, no research has been published so far on the administration of I curly endive biofortification in the human healthy population. Starting from the above-mentioned premise, this study aimed to assess the potential effects of short nutritional intervention with I biofortified curly endive on a population of healthy individuals.

## Materials and Methods

### Trial Setup, Plant Materials, Nutraceutical Traits, and Crop Management

**FIGURE 1:**
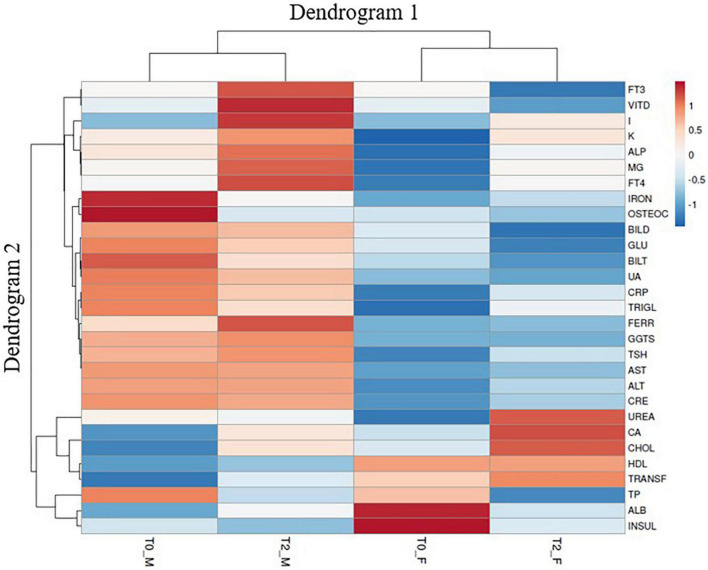
Flowchart of the interventional nutritional study during the period of 12 days of administration of biofortified iodine curly endive and curly endive without any biofortification. Created with “BioRender.com”.

In this study, I-enrichment was made by supplying I in the form of potassium iodate provided through foliar spray during the period of growth. After the harvesting season, the endive crops were provided for human consumption in a selected population. In detail, 100 g of curly endive was consumed by a cohort of twenty individuals, and blood and urine samples were obtained at the beginning and after 12 days of consumption (Figure 1). With regard to I determination, the I content in leaf tissues and urine samples was assessed *via* inductively coupled plasma mass spectrometry (ICP-MS). The crop was cultivated following the work of Sabatino et al. (14).

### Subject Recruitment and Study DESIGN

A cohort of 20 healthy subjects (10 men and 10 women) of different ages (age range 20–64) (Table 1) was recruited at the Policlinico Hospital “Paolo Giaccone Palermo.” The healthy volunteers were all Sicilians, living in the area of West Sicily. A group of well-trained nutritionists and physicians administered a questionnaire to collect anamnestic data of interest, food habits (24h recall), and lifestyle. Participants were selected on the basis of their health status since none of them had neoplastic, infective, or autoimmune diseases, and none were prescribed drugs known to interfere with immune-inflammatory responses. No antibiotics, vitamins, or minerals were used for >6 weeks before the beginning of the study. The women were not using exogenous hormones. The volunteers were advised to not use any product containing I, such as disinfectant, dietary supplements (iodized salt), beauty cream for weight loss, or drugs, and they do not undergo radiological procedures using iodinated contrast media. At the beginning of the 12-day study and at the end of the study period, thyroid volume and urinary iodine (UI) excretion were measured (data not shown). We advised the cohort to follow the same regular nutritional pattern.

**TABLE 1:** Characteristics of the subjects.

Participants	Male *n* = 10	Female *n* = 10
Age (years)	41.6 ± 9.9	46.6 ± 4.8
Weight (Kg)	87.9 ± 12.1	65.6 ± 3.1

*Values are presented as mean and SD.*

Participants signed informed consent before the enrolment. To respect privacy, everyone was identified with an alphanumeric code. A database was created to handle the collected information. The study protocol, conducted in accordance with the Declaration of Helsinki and its amendments, was approved by the Ethics Committee of Palermo University Hospital (Biofortification, No.2/2020) AIFA CE 150109. The trial was registered at Clinicaltrials.gov NCT05011968 (Nutr-I-Food2019). All methods were performed in accordance with the relevant guidelines and regulations.

In detail, three entire curly endive plants (in total at least 2 kg) were given to each participant in order to utilize 100 g every day for a period of 12 days. Plants were stored at 4 ± 1°C. Healthy volunteers were divided into two groups, namely, 10 received control curly endive and 10 received iodinated curly endive. Both cohorts were asked to write a food diary during the study.

At baseline (T0) and after 12 days (T2), blood and urine samples were obtained. The healthy volunteers were advised to not use any food supplementation or integration 10 days before the baseline and for the entire period of 12 days of curly endive administration.

The recruited participants underwent vein puncture, after a fasting period of 10–12 h. The fasting blood samples were obtained in the morning (between 8.30 and 10 a.m.) and were collected in serum tubes with no additives. Hematochemical tests, carried out for all subjects, were performed at the Central Laboratory Analysis of Palermo University Hospital according to the standard procedure at baseline (T0) and after 12 days (T2) (Figure 1). Blood measurement regards potassium (K), albumin (ALB), alkaline phosphatase (ALP), alanine transaminase (ALT), calcium (Ca), creatinine (CRE), uric acid (UA), ferritin (FER), high-density lipoproteins (HDL) cholesterol, transferrin (TRAN), reactive C protein (RCP), cholesterol (CHO), glucose (GLU), magnesium (MG), urea (UREA), triiodothyronine (FT3), thyroxine (FT4), thyroid-stimulating hormone (TSH), bilirubin direct (BILD), bilirubin indirect (BILT), gamma-glutamyl transferase (GGTS), insulin (INSUL), osteocalcin (OSTEOC), iron (IRON), total protein (TP), triglycerides (TRIGL), and vitamin D (VITD). Urine samples were collected in the morning (between 8.30 and 10 a.m.) in falcon tubes and stored at −80°C

### I Determination and Calibration

To analyze I content, air-dried lettuce samples were ground in a variable speed rotor mill Pulverisette 14 FRITSCH (Idar-Oberstein, Alemania, Germany) using a 0.5-mm sieve. Digestion of 0.5 g samples of lettuce in the mixture 348 Rakoczy et al. of 10 cm^3^ 65% HNO_3_ and 0.8 cm^3^ 70% HClO_4_ was conducted in the microwave system MARS-5 Xpress (CEM, World Headquarters, Matthews, NC, United States). The content of I was analyzed by the cold vapor generation technique with the use of high-dispersion inductively coupled plasma optical emission spectrometry (ICP-OES, Prodigy spectrometer, Leeman Labs, United States). In urine, the content of I in these samples was analyzed by the cold vapor generation technique with the use of ICP-OES (15, 16) after sample digestion in the mixture of 10 cm^3^ 65% HNO_3_ and 0.8 cm^3^ 70% HClO_4_ in the microwave system CEM MARS-5 Xpress. To analyze I in urine, samples were thawed, mixed, and diluted 10 times before the analysis without any digestion step or oxidation. Calibration standard solutions for iodate were prepared on a daily basis by stepwise dilution of the KIO_3_ solution in a 1% HNO_3_ medium to yield a final concentration of 20, 30, 40, 50, 75, 100, and 250 μg/L. The solution containing I^+^ (50 μg L^–1^) was used as internal standards to compensate for any signal instability or sensitivity changes during the analysis. A solution of 2% HNO_3_ as blank was used. The I^+^ ion was measured at m/z 127. The detection limit was determined as 3 SDs of the concentration in the urine sample.

### Experimental Designs and Statistics

The healthy volunteers attended the first visit and underwent anthropometric measurements (Table 1) and 24 dietary recalls. Daily nutritional intake was recorded over a period of 8 consecutive days before starting the study and until the end of the experiment (food diary). This first period was assumed to be representative of the healthy volunteer’s habitual nutritional intake. The healthy volunteers were provided with a food diary and instructed to record all food and beverages (including quantities) consumed over the 8 days before starting the study and until the end of the study. The blood sample (baseline sample) was collected prior to the experimental condition start. Student *t*-tests were used to compare the baseline characteristics of the two groups. Changes between baseline and follow-up within the groups were analyzed using paired *t*-tests using the GraphPad Prism program. A heat map that sums up clinical parameter responses to the human consumption of I-biofortified curly endive was also created using the https://biit.cs.ut.ee/clustvis/ program package with Euclidean distance as the similarity measure and hierarchical clustering with complete linkage.

## Results

### I Concentration in Leaf Tissues

For plant I concentration, the highest curly endive leaf tissue I concentration was detected in plants enriched with 250 mg I L^–1^ grown in the fall season (241.97 mg kg^–1^ dry weight), those plants were used for the nutritional intervention, and the concentration was confirmed *via* ICP-MS (14).

### Participants, Study Design, and Compliance

The short pilot interventional study involved 20 volunteers aged 20–64 years (10 women and 10 men). All participants were in good general health, and during the short period of I curly endive administration, there was no evidence of weight modification (Table 1). All the participants ended the short-time nutritional intervention after 12 days without any drop-out and best compliance.

### Urinary and Hematological Parameters

Biofortification with I did not affect the whole body homeostasis in healthy people. In fact, I, glucose, lipid, hepatic, and iron metabolism did not change after 12 days of consumption of I biofortified curly endive either in the control group or in the iodinated group (Table 2). Moreover, the assumption for 12 days of 100 g of I curly endive resulted in a statistically significant increase of vitamin D (*P*-value 0.039), calcium (*P*-value 0.0007), and potassium (*P*-value 0.0097) measurements in blood samples with respect to control (Table 2). Furthermore, urinary samples show a significant increase in I concentration (*P*-value 0.04) after 12 days of I curly endive administration with respect to control (Table 2). Data on I absorption ranged from 20.61 mg day^–1^ (99.0% of the I intake *via* biofortified curly endive consumption) to 20.72 mg day^–1^ (99.6% of the I intake *via* biofortified curly endive consumption) for men and women, respectively. Although, ANOVA pointed out that sex did not influence I absorption.

**TABLE 2:** Hematological and urinary parameters.

	Iodine lettuce					Control lettuce				
Blood parameters	T0	SD	T2	SD	*p*-value	T0	SD	T2	SD	Range
K (mmol/L)	4.3	0.5	4.6	0.4	**0.0097**	4.1	0.6	4.1	0.4	3.5–5.1
ALB (mg/dL)	45.3	3.2	44.8	3.3		43.7	5.6	44.8	3.6	35–52
ALP (U/L)	54.5	10.3	60.4	10.0		56.3	7.9	54.5	9.3	40–129
ALT (U/L)	21.1	13.9	23.9	11.0		21.1	9.2	21.5	8.5	0–41
AST (U/L)	19.6	5.4	19.9	5.0		19.5	7.2	20.1	7.3	0–37
BILD (mg/dL)	0.3	0.1	0.2	0.1		0.3	0.1	0.3	0.1	<0.3
BILT (mg/dL)	0.7	0.5	0.5	0.3		0.6	0.4	0.5	0.4	<1.2
CA (mg/dL)	9.4	0.2	9.7	0.4	**0.0007**	9.1	0.5	9.1	0.4	8.6–10.2
CHO (mg/dL)	181.8	25.6	187.2	13.8		178.1	22.7	170.2	23.1	0–200
CRE (mg/dL)	0.8	0.2	0.8	0.1		0.7	0.2	0.8	0.1	0.67–1.17
CRP (mg/L)	0.8	0.7	0.9	0.7		0.9	0.5	1.1	0.6	0–0.5
FERR (mg/dL)	93.7	81.9	120.4	96.8		116.7	79.3	113.6	67.3	30–400
GGTS (U/L)	22.6	30.7	23.9	32.0		28.5	25.8	32.2	23.6	8–61
GLU (mg/dL)	91.7	10.8	89.9	8.2		79.7	6.5	80.6	16.8	70–115
HDL (mg/dL)	60.1	19.9	61.8	16.6		54.5	7.9	52.1	16.6	35–55
IRON (mg/dL)	81.2	22.5	78.9	25.3		83.3	8.0	76.5	14.7	33–19
MG (mg/dL)	1.9	0.2	2.0	0.1		1.7	0.2	1.7	0.4	1.6–2.5
TP (mg/dL)	73.9	4.6	71.2	4.3		67.3	8.6	67.3	8.6	66–87
TRICL (mg/dL)	76.1	40.9	78.5	33.7		73.5	22.0	76.5	38.7	0–200
TRAN (mg/dL)	252.6	50.0	269.2	33.4		186.6	78.6	213.8	58.4	200–360
UA (mg/dL)	5.5	1.6	5.1	1.5		5.8	2.2	5.4	1.6	3.4–6.5
UREA (mg/dL)	30.1	6.4	34.2	7.6		25.8	9.3	25.7	7.3	10–50
FT3 (pmol/L)	3.0	0.2	3.0	0.4		2.8	1.2	2.9	0.8	2–44
FT4 (pmol/L)	1.1	0.1	1.1	0.1		1.3	0.9	1.1	0.4	0.70–1.7
INSUL (mg/dL)	18.0	22.1	15.2	16.7		14.6	8.3	15.1	9.4	2.4–24.6
OSTEOC (ng/mL)	22.9	7.8	20.7	3.4		19.5	8.2	19.8	7.2	11–46
TSH (pmol/L)	2.0	0.9	2.4	0.7		2.7	1.4	2.5	1.4	2.27–4.2
VIT D (ng/L)	2.3	3.3	26.8	5.5	**0.039**	22.6	3.8	21.2	2.1	>30
Urinary parameters										
I (μg/L)	133.4	70.2	282.1	176.1	**0.04**	136.7	64.4	163.0	105.4	>200

*SD, standard deviation. Values are means ± SD. n = 10 in each group and statistical significance is set at P < 0.05.*

*(K), potassium; (ALB), albumin; (ALP), alkaline phosphatise; (ALT), alanine transaminase; (Ca), calcium; (CRE), creatinine; (UA), uric acid; (FER), ferritin; (HDL) high-density lipoproteins; (TRAN), cholesterol transferrin; (RCP), reactive C protein; (CHO), cholesterol; (GLU), glucose; (MG), magnesium; (UREA), urea; (FT3), triiodothyroinine; (FT4), thyroxine; (TSH), thyroid stimulating hormone; (BILD), biliribin direct; (BILT), biliribin undirect; (GGTS), gamma-glutamyl transferase; (INSUL), insulin; (OSTEOC), osteoclacin; (IRON), iron; (TP), total protein; (TRIGL), triglycerides; (VITD), vitamin D.*

### Heat Map Analysis of All Urinary Clinical Parameters

**FIGURE 2:**
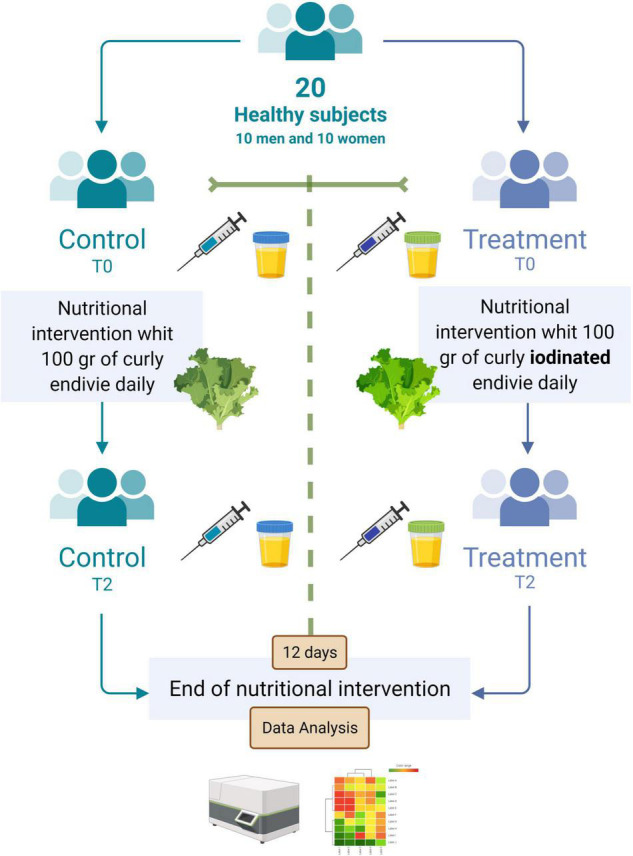
Cluster heat map analysis summarizing the blood and urinary parameter response to a factorial experiment with two sampling times, namely, T0 and T2 (T0 = baseline and T2 after 12 days post-biofortification) and gender M and F (F = female, M = male). The figure was generated using the https://biit.cs.ut.ee/clustvis/ program package with Euclidean distance as the similarity measure and hierarchical clustering with complete linkage. The statistical software attributed colors from red to blue as the values of the measurement decrease. From blue to red as the values increase.

A clustered data heat-map analysis of all urinary and hematological traits was conducted to display a chromatic evaluation of the different combination of sex × time of clinical analysis (pre- or post-biofortification) (Figure 2). The heat map analysis exhibited two dendrograms; Dendrogram 1, planned on the top, is a classification that matched to the combinations of sex × time of clinical analysis (T0_M, T2_M, T0_F, and T2_F) and the second dendrogram, named Dendrogram 2, showing the features that influenced this distribution. Dendrogram 1 revealed two main clusters: on the left, the cluster corresponded to male sex for each time of clinical analysis (pre- or post-biofortification), whereas on the right of the heat map, the cluster comprises the T0_F and T2_F combinations (Figure 2). Specifically, on the left of Dendrogram 1, two groups were recognized. The first on the left includes the combination T0_M, separated from T2_M, which exposes particularly lower values for IRON, OSTEOC, GLU, BILT, UA, CRP, TRIGL, TP, and INSUL but higher values for FT3, VITD, I, K, ALP, MG, FT4, FERR, GGTS, TSH, CA, CHOL, TRANSF, and ALB.

The right side of Dendrogram 1 revealed two groups, the first on the left including T0_F separately from T2_F, which displayed, exactly, higher I, K, ALP, MG, FT4, IRON, CRP, TRIGL, TSH, ALT, CRE, UREA, CA, CHOL, and TRANSF. The group T2_F showed lower values than T0_F for FT3, VITD, OSTEOC, BILD, GLU, BILT, UA, and TP. Attractively, the clusters in Dendrogram 2 evidently pointed out the diverse influences of the different combinations of sex × time of clinical analysis (pre- or post-biofortification).

## Discussion

Iodine is an essential trace element for the biosynthesis of thyroid hormones and a key element of different metabolic pathways in human (24, 25). I shortage is caused by inadequate dietary I consumption and determines various types of illnesses.

The WHO has drawn attention to mineral deficiency analyzing that approximately 45% of European people are spanning out the physiological requirements of I.^1^ Therefore, the “Recommended Daily Allowance” (RDA) for I is estimated around 90–120 μg for children, 150 μg for adults, and 290 μg for pregnant or breastfeeding women as reported by the European Food Safety Authority (EFSA NDA Panel, 2014) (19).

The WHO’s classifications of I nutritional status are based on UIC values of <20 μg/L (severe I deficiency), 20–49 μg/L (moderate I deficiency), 50–99 μg/L (mild I deficiency), 100–199 μg/L (adequate I intake), 200–299 μg/L (more than adequate I intake), and >300 μg/L (excessive I intake) (26). UIC levels of >300 μg/L are also associated with risks of I-induced goiter, hyperthyroidism, and hypothyroidism (27).

The present short-term pilot nutritional intervention study evaluated the potential beneficial effect of 12 days of supplementation with curly endive crops fortified with 250 mg L^–1^ of IO_3_^–^ (iodate of potassium) in healthy volunteers, enrolled according to our inclusion and exclusion criteria. In this short pilot study, we have found that consumption of 100 g of curly endive for 12 days increases urinary I excretion in a considerable manner. I supplementation seems to show a positive influence on vitamin D concentration, calcium, and potassium. Our first hypothesis is that I biofortified lettuce is the perfect matrix to permit the physiological absorption and regulation of the different minerals. However, this is not the only reason for the results that we showed since curly endive without I biofortification did not achieve the same results.

One possible hypothesis, on I and vitamin D association, comes from several studies, which have found a correlation between the incidence of autoimmune thyroid disease and vitamin D deficiency (28, 29). Several human studies showed that patients with hypothyroidism often exhibited lower levels of vitamin D or vitamin D deficiency with respect to healthy controls. On the contrary, correlations between vitamin D concentrations and antibodies against peroxidase (TPOAb), thyroglobulin (TgAb), and TSH in patients with hypothyroidism seem to exist as well as a positive relationship of vitamin D with T3 levels (30). Looking inside the I vs. vitamin D association, we saw that I seems influencing gut microbiota composition (31), and gut microbiota plays a key role in the thyroid-gut-axis regulating vitamin absorption (32). Besides, the gut microbiota is effective on I absorption and thyroid hormone homeostasis due to deiodinase activity in the intestinal wall (33). In both cases, there is a strong correlation between vitamin D and I, but we still cannot define who does what at first hand. Regarding vitamin D and calcium, those are strictly correlated as well as vitamin D and potassium, although potassium level might be related to the salt (iodate of potassium) used for biofortification. Nevertheless, potassium levels in the blood are influenced by adrenal glands, which regulate potassium concentration *via* kidney excretion (34) and vitamin D impact on kidney function. Furthermore, vitamin D has potential implications for mitochondrial bioenergetics and cytoprotective mechanisms working on the K channel (35).

From the physiological point of view, potassium concentration that is able to stimulate bone resorption and interventional studies in human showed that low potassium concentration induces calcium excretion that in turn impacts bone remodeling. Thus, vitamin D, calcium, and potassium are involved in the action mechanism that improves bone turnover (36, 37). In addition, it is known that thyroid hormones have a strong effect on bone metabolism and osteoporosis (38).

In our experiment, we saw an increase in I urine levels probably due to I biofortified food consumption. It is worthy to note that the amount of I in biofortified curly endive consumed corresponds to 1,210 μg of I/100 of fresh weight (with 5% of dry matter).

This value is very high if considered that the RDA for this mineral element is 150 μg/day. However, despite this, no negative effects were found after the short-term nutritional intervention. In addition, our results showed an increase in vitamin D and calcium, while an increase in potassium probably comes from the salt used to complex I. So, I, vitamin D, calcium, and potassium can be the players in bone homeostasis and bone formation (39, 40). Furthermore, vitamin D has a paramount role both in innate and adaptive immunity, and there is strong indication supporting its involvement in many other “non-classical” disorders not related to the bones, such as cancer, cardiovascular, metabolic disorders, multiple sclerosis, and infectious diseases (41). Furthermore, I supplementation can be a valuable solution for the market of biofortified vegetables (42), and human intervention trials are mandatory since there are scares in literature (43–46).

In addition, blood analysis has shown that I biofortification does not alter FT3, FT4, and TSH values and does not affect glucose, lipid, hepatic, and iron metabolism. From the gender point of view, the clusters in Dendrogram 2 evidently pointed out the diverse influences of the different combinations of sex × time of clinical analysis (pre- or post-biofortification), while ANOVA analysis shows only I concentration associated with consumption of I-biofortified curly endive.

Nonetheless, our study presents some limitations as follows: the small sample size; the interindividual/intraindividual variability; and the short duration of the intervention. However, the longer the duration of the intervention, the greater the drop-out effect. Although the strength of this kind of study is the ability to link the outcome to the intervention, thanks to the double control (pre and post) and the second arm as a control cohort, it is not possible to monitor compliance in the absence of a specific marker. So, the linkage to the intervention is probably but not fully attributed.

## Conclusion

Food fortification is a policy that has been used carefully and successfully to prevent micronutrient deficits in high-income countries for some time (47). The relationship between biofortified food intake and human health is a contemporary and innovative research matter. In this regard, findings reveal that food compounds, especially in biofortified food, influence the physiological responses of the human body. Accordingly, functional foods are very interesting and promising to prevent and cure diverse human disorders.

In this short-term nutritional intervention study, we proposed that I enrichment curly endive crops might be of beneficial effect on achieving healthy range micronutrient concentration in the human population. Furthermore, the increase in calcium, potassium, and vitamin D concentration might have beneficial influences either on bone homeostasis or non-classical bone diseases.

In the field of the functional food market, nutrient supplementation *via* vegetables or fruit carriers seems to have better bioavailability, absorption, and compliance with respect to pill supplementation, although further nutritional intervention studies are warranted. In conclusion, the production of biofortified vegetables will deserve a noticeable place in the coming decades.

## Data Availability Statement

The original contributions presented in the study are included in the article/Supplementary Material, further inquiries can be directed to the corresponding authors.

## Ethics Statement

The studies involving human participants were reviewed and approved by Ethic Committee of Palermo University Hospital (Biofortification, No.2/2020) AIFA CE 150109. The trial is registered at Clinicaltrials.gov NCT05011968 (Nutr-I-Food2019). The patients/participants provided their written informed consent to participate in this study.

## Author Contributions

SB and LD performed the statistical analyses and interpreted the data. SV drafted the manuscript. FD and SB provided substantial contributions to the idea, conception, and design of the study, interpretation of the data, and guidance on the intellectual content, and critical revision of the manuscript. LD, DN, PP, and CDP contributed to the critical revision of the manuscript. RC contributed to the revision of the manuscript. All authors have read and agreed to the published version of the manuscript.

## Conflict of Interest

The authors declare that the research was conducted in the absence of any commercial or financial relationships that could be construed as a potential conflict of interest.

## Publisher’s Note

All claims expressed in this article are solely those of the authors and do not necessarily represent those of their affiliated organizations, or those of the publisher, the editors and the reviewers. Any product that may be evaluated in this article, or claim that may be made by its manufacturer, is not guaranteed or endorsed by the publisher.

## Footnotes

1
http://who.int/vmnis/database/iodine/en

## Supplementary Material

Supplementary Figure 1 Graphical abstract showing the different phases of biofortified food production, administration and results processing.
