# Supplementary figures and images for: Biofortification: Effect of Iodine Fortified Food in the Healthy Population, Double-Arm Nutritional Study
Source: Front Nutr. 2022 Mar 23;9:871638. doi: 10.3389/fnut.2022.871638 (PMC8984677; doi:10.3389/fnut.2022.871638)

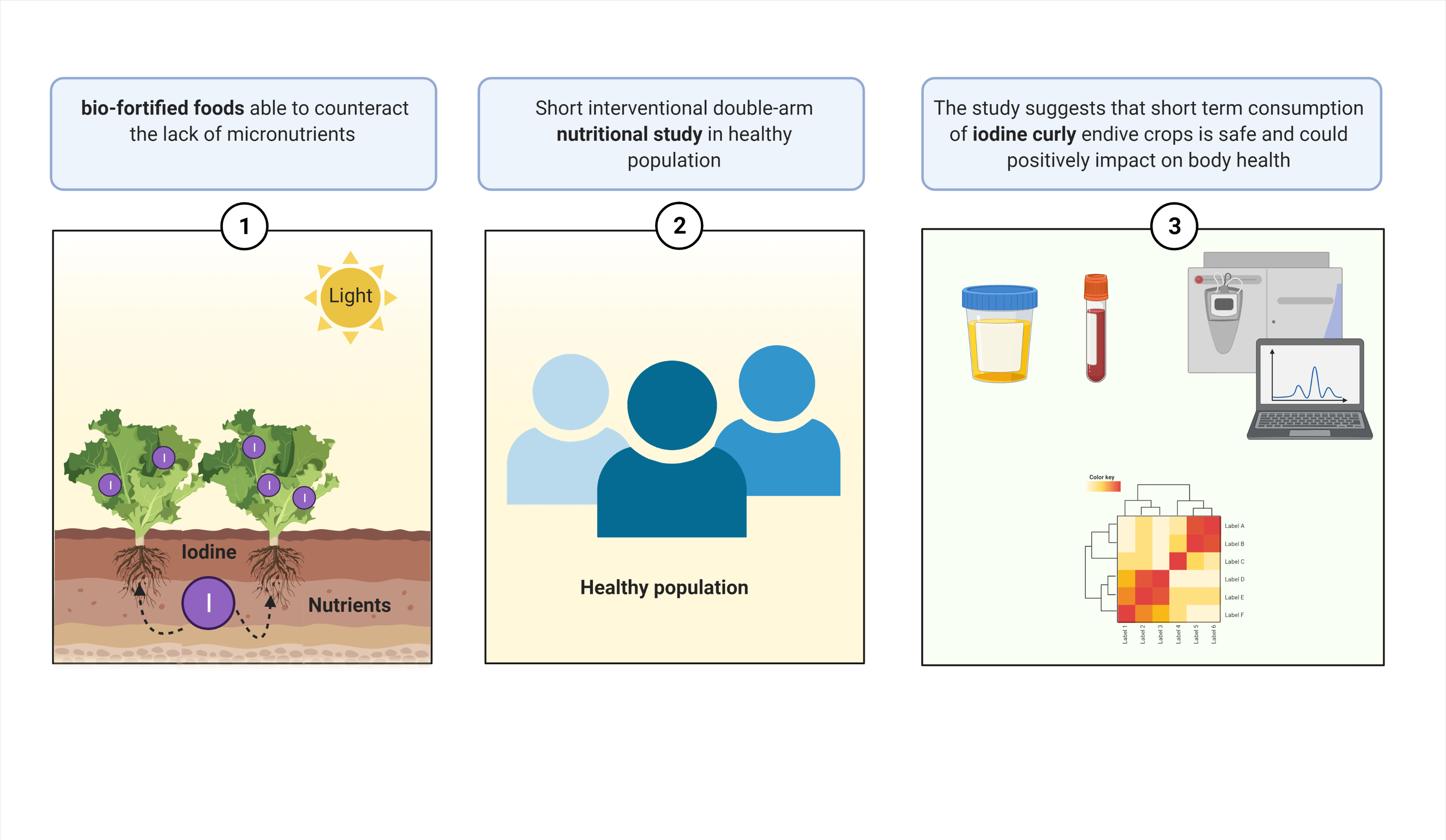

Supplement: Supplementary Figure 1 — Graphical abstract showing the different phases of biofortified food production, administration and results processing. [file Image_1.jpeg]
